# Oxidized high‐density lipoprotein associates with cardiometabolic dysfunction in coronary artery disease and acute coronary syndrome

**DOI:** 10.1111/joim.70019

**Published:** 2025-09-10

**Authors:** Benjamin Sasko, Nikolaos Pagonas, Martin Christ, Jan Wintrich, Oliver Ritter, Christian Ukena, Innas Sultana, Simin Delalat, Ibrahim El‐Battrawy, Theodoros Kelesidis, Nazha Hamdani

**Affiliations:** ^1^ Medical Department II Ruhr University Bochum, Marien Hospital Herne Herne Germany; ^2^ University Medical Center Brandenburg an der Havel, Medical School Theodor Fontane Brandenburg an der Havel Germany; ^3^ Department of Cardiology University Hospital Ruppin‐Brandenburg, Medical School Theodor Fontane Neuruppin Germany; ^4^ Faculty of Health Sciences Joint Faculty of the Brandenburg University of Technology Cottbus—Senftenberg the (MHB) Theodor Fontane and the University of Potsdam Potsdam Germany; ^5^ Department of Cardiology Knappschaftskrankenhaus Bottrop Academic Teaching Hospital University Duisburg‐Essen Bottrop Germany; ^6^ Department of Cellular and Translational Physiology Institute of Physiology Ruhr University Bochum Bochum Germany; ^7^ Institut für Forschung und Lehre (IFL) Molecular and Experimental Cardiology Ruhr University Bochum Bochum Germany; ^8^ Department of Cardiology and Rhythmology St. Josef Hospital Ruhr University Bochum Bochum Germany; ^9^ Department of Medicine Division of Infectious Diseases and Geographic Medicine University of Texas Southwestern Medical Center Dallas Texas USA; ^10^ Department of Pharmacology and Pharmacotherapy Center for Pharmacology and Drug Research & Development HCEMM‐SU Cardiovascular Comorbidities Research Group Intézet Címe Semmelweis University Budapest Hungary Budapest Hungary; ^11^ Department of Physiology Cardiovascular Research Institute Maastricht Maastricht University Maastricht The Netherlands

**Keywords:** acute coronary syndrome, cardiometabolic function, HDL function, oxidative stress, oxidized HDL

## Abstract

**Background:**

High‐density lipoprotein (HDL) function, rather than its concentration, plays a crucial role in the development of coronary artery disease (CAD). Diminished HDL antioxidant properties, indicated by elevated oxidized HDL (nHDL_ox_) and diminished paraoxonase‐1 (PON‐1) activity, may contribute to vascular dysfunction and inflammation. Data on these associations in CAD patients, including acute coronary syndrome (ACS), remain limited. The aim of this study is to assess the association of oxidized HDL with PON‐1 activity, oxidized low‐density lipoprotein (LDL), vascular cell adhesion molecule‐1 (VCAM‐1), IL‐6 levels, and nitric oxide (NO) production as markers of vascular health.

**Methods:**

We assessed HDL function in three groups: 90 CAD patients, 90 healthy controls, and 90 ACS patients. HDL antioxidant function was measured using a validated biochemical assay to quantify oxidized HDL (nHDL_ox_). Plasma PON‐1 activity, oxidized LDL, VCAM‐1, IL‐6, and NO production were also evaluated.

**Results:**

ACS patients had nHDL_ox_ levels 140% higher than healthy controls (*p* < 0.001). Higher nHDL_ox_ levels were significantly linked to vascular inflammation, reflected by elevated VCAM‐1 levels. Additionally, a reduced PON‐1 activity indicates an impaired antioxidant protection in ACS patients. Finally, oxidized LDL levels were elevated, and NO production was reduced, suggesting impaired vascular function.

**Conclusion:**

HDL_ox_ levels are highest in patients with ACS. Patients with stable CAD have higher levels than healthy controls. Correspondingly, the parameters of HDL function measured in this study, which all indicate a loss of HDL's atheroprotective function, correlate with these findings. Our study establishes a novel mechanistic pathway linking oxidized HDL to the presence of an ACS.

**Clinical trial registration:**

DRKS00014037

## Graphical abstract

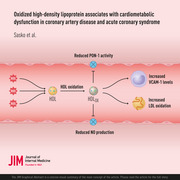

**Abbreviations**
ACS: acute coronary syndrome
BMI: body mass index
CAD: coronary artery disease
CVD: cardiovascular disease
HDL: high‐density lipoprotein
HDL_ox_
: HDL‐lipid peroxide content
HFpEF: heart failure with preserved ejection fraction
IL‐6: Interleukin‐6
LDL: low‐density lipoprotein
LDL_ox_
: oxidized low‐density lipoprotein
nHDL_ox_
: normalized HDL‐lipid peroxide content
NO: nitric oxide
PON‐1: paraoxonase‐1
VCAM‐1: vascular cell adhesion molecule‐1

## Introduction

Atherosclerotic cardiovascular disease (CVD) remains a major cause of morbidity and mortality in the world, with coronary artery disease (CAD) playing a crucial role in this challenge. Despite extensive research, the mechanisms that contribute to CAD pathogenesis remain partly unresolved. High‐density lipoprotein cholesterol (HDL‐C) is generally considered a protective factor against CVD, with its levels serving as crucial indicators of cardiovascular risk. Nevertheless, new research suggests that HDL function, rather than HDL‐C levels alone, can provide a more accurate assessment of cardiovascular health [1, 2]. The anti‐atherosclerotic roles of HDL include promoting cholesterol efflux [3], enhancing endothelial function [4, 5], and exerting anti‐inflammatory [6, 7, 8] and antioxidant effects [9, 10, 11].

Nevertheless, HDL can become dysfunctional under inflammation and oxidative stress conditions, thereby losing its protective properties and contributing to an increased risk of CVD [12, 13, 14]. This phenomenon underscores the complexity of HDL's role in atherogenesis, particularly in oxidative stress and oxidized HDL (HDL_ox_) formation [15, 16]. Several studies have demonstrated that HDL oxidation contributes to the formation of dysfunctional HDL [14, 15], and HDL oxidative properties are closely related to the functional properties of HDL [16].

HDL dysfunction is denoted by reduced cholesterol efflux capacity and compromised inhibition of low‐density lipoprotein (LDL) oxidation [9, 12, 17, 18]. As HDL's protective functions are critical to mitigating oxidative, inflammatory, and thrombotic mechanisms, the emergence of dysfunctional HDL may play a pivotal role in the pathophysiology of CAD. Numerous studies have linked HDL dysfunction with a variety of CVDs, including CAD [17, 18], ischemic cardiomyopathy [19], atrial fibrillation [20], and heart failure [21, 22]. Furthermore, HDL_ox_ correlates with acute cardiovascular events [23] and foam‐cell formation [24].

The oxidative modification of HDL diminishes its protective functions and may transform it into a pro‐atherogenic entity. HDL's antioxidant properties, primarily mediated by enzymes such as paraoxonase‐1 (PON‐1), are crucial in scavenging reactive oxygen species (ROS) and preventing LDL oxidation [16]. When HDL becomes oxidized, PON‐1 activity declines, reducing HDL's ability to counteract oxidative stress [25]. This dysfunctional, antioxidant‐deficient form of HDL contributes to a cascade of pathological processes that promote atherogenesis. Endothelial dysfunction, caused by HDL oxidation, is characterized by decreased nitric oxide (NO) bioavailability, enhanced vascular resistance, and heightened vascular inflammation. These alterations compromise vascular health and accelerate the formation of atherosclerotic plaques.

The pro‐inflammatory effects of oxidized high‐density lipoprotein (HDL_ox_) further exacerbate atherogenesis. In contrast to healthy HDL, which reduces the expression of vascular cell adhesion molecule‐1 (VCAM‐1) and prevents leukocytes from adhering to the endothelium, the oxidized HDL loses its ability to bind to the scavenger receptor class B Type 1 (SR‐B1) and, paradoxically, promotes endothelial inflammation [26, 27]. Oxidized HDL stimulates the expression of VCAM‐1 and activates nuclear factor‐κB (NF‐κB), resulting in enhanced leukocyte recruitment and increased vascular inflammation. This increase in pro‐inflammatory activity promotes the disruption of atherosclerotic plaques and accelerates the progression of CAD. Additionally, the loss of anti‐apoptotic properties in HDL_ox_ exacerbates endothelial cell damage. Normal HDL inhibits apoptosis by suppressing caspase‐3 activation and enhancing endothelial NO synthase (eNOS) activity [28]. However, oxidized HDL fails to provide these protective effects, increasing endothelial cell apoptosis and subsequent vascular injury.

Due to HDL_ox_’s pivotal involvement in atherogenesis, there is growing interest in exploring its potential as a biomarker for cardiovascular risk. However, the precise role of HDL_ox_ in the progression of CVD is still not fully understood. This study aims to elucidate the correlation between HDL_ox_ and PON‐1 activity, VCAM‐1 levels, NO production, and oxidized low‐density lipoprotein (LDL_ox_) in a cohort of patients with stable CAD, acute coronary syndrome (ACS), and healthy controls. By evaluating the relationships between HDL_ox_ and these key markers of vascular health, the study seeks to advance our understanding of the mechanisms driving atherogenesis and identify potential therapeutic targets to restore HDL functionality.

## Methods

### Study

This study was conducted at the University Hospitals of Brandenburg and Bochum and included three cohorts (CAD, ACS, and controls), each comprising 90 participants randomly selected from a previously described cohort [18]. Patient recruitment and inclusion/exclusion criteria have been detailed in earlier publications [18, 20]. Briefly, CAD and control participants were enrolled during elective coronary angiography between 2017 and 2019, whereas ACS patients underwent urgent angiography between 2019 and 2021. Only Type 1 myocardial infarction cases were included; patients with COVID‐19, Type 1 diabetes, cancer, acute infections, rheumatic disease, or under 18 years were excluded.

All participants provided written informed consent. The study was approved by the local ethics committees of the Medical Association of Brandenburg (Nr. AS69bB/2016) and the Ruhr University of Bochum (Nr. 15‐5279) in accordance with the Declaration of Helsinki. The data supporting the findings of this study are available from the corresponding author upon reasonable request.

### Biomarker and laboratory assessment

Fasting blood samples were collected before any procedures in the elective patients or within 48 h after admission in ACS (mainly after coronary angiography). Isolated serum from blood samples was cryopreserved (−80°C). Grossly hemolyzed serum was excluded from further analysis.

Standard clinical assays in the central laboratory unit of the university hospitals obtained laboratory parameters. High‐sensitivity C‐reactive protein (hs‐CRP) was measured using the Roche Tina‐Quant CRP (Latex) kit (Roche Diagnostics, Basel, Switzerland). According to the manufacturer's instructions, the lipoprotein‐associated phospholipase A2 (Lp‐PLA2) was determined by the Diasys Lp‐PLA2 activity reagent (Diasys Diagnostic Systems, Germany).

### Assessment of lipid peroxide content of HDL (HDL_ox_)

HDL_ox_ levels in serum were measured using a modified version of a validated fluorometric biochemical cell‐free assay. This method detects HDL lipid peroxide content by tracking the oxidation of the fluorochrome Amplex Red [29, 30]. Initially, polyethylene glycol (PEG) precipitation was used to deplete apolipoprotein B (ApoB) from serum. After adding 50 µL of ApoB‐depleted serum to each well of a 96‐well plate in duplicate, 0.075 U of horseradish peroxidase (HRP) plus 50 µM Amplex Red reagent were applied to each well to reach the total volume of 100 µL. HRP catalyzes the reaction between Amplex Red and resorufin when it is coupled with endogenous peroxides. After 1 h of incubation, a Spark 10M microplate reader (Tecan, Austria) was utilized to determine the fluorescence of resorufin at 535/590 nm wavelengths. To normalize and minimize experimental variability across each plate, ApoB‐depleted sera from 10 healthy volunteers (who were not participants in the study) were employed as experimental controls. Mean fluorescence from each sample was normalized by the mean fluorescent readout of the pooled control and HDL‐C using the following calculation: “normalized” oxidized HDL (nHDL_ox_) = [HDL_ox__sample × 47 (mg/dL)]/[HDL_ox__control × HDL‐C sample (mg/dL)], where 47 mg/dL represents HDL‐C of the pooled serum control. Samples were analyzed after the recruitment of the last patient. The intra‐assay coefficient of variation (CV) was 6.7%. The inter‐assay CV was 3.7%. All laboratory measurements were performed in Brandenburg, Germany.

### Assessment of paraoxanase‐1 (PON‐1) activity

PON‐1 activity was assessed utilizing the Human PON‐1 ELISA Kit (Thermo Fisher, Cat. No. EH376RB) as per the manufacturer's guidelines. Briefly, serum samples were collected in pyrogen/endotoxin‐free tubes, frozen immediately, and thawed before analysis. Serum and plasma samples were diluted 20 times in 1X Assay Diluent. The assay was conducted in a 96‐well plate precoated with anti‐human PON‐1 antibody, followed by incubation with biotin‐conjugated PON‐1 antibody and Streptavidin‐HRP. Following a washing step, TMB substrate was applied, and Stop solution was used to stop the reaction. Absorbance was quantified at 450 nm, and PON‐1 concentrations were determined using a four‐parameter standard curve. The intra‐ and inter‐assay measures’ coefficients of variation, which were less than 10% and 12%, respectively, guaranteed assay accuracy.

### Assessment of vascular cell adhesion molecule‐1 (VCAM‐1)

Serum levels of VCAM‐1 in controls, CAD, and ACS were quantified utilizing the Invitrogen Human VCAM‐1 ELISA Kit (Thermo Fisher Scientific, KHT0601), according to the manufacturer's protocol. To summarize, diluted serum samples and standards were introduced into a pre‐coated 96‐well plate, followed by incubation with a biotinylated detection antibody and streptavidin‐HRP. The enzymatic reaction was subsequently developed using TMB substrate and terminated with Stop solution. Absorbance readings were taken at 450 nm, and concentrations were calculated using a four‐parameter logistic (4PL) standard curve. All samples were analyzed in duplicate.

### Assessment of interleukin‐6 (IL‐6)

To quantify IL‐6 in serum from controls, CAD, and ACS, we used an ELISA kit from Thermo Scientific according to the structure of the manufacturer's instructions with minor modifications. Following the equilibrium to the room temperature and a gentle mixing, samples were kept on the ice. Further, biotinylated antibody reagents and serum samples were added to the wells, followed by incubation and washing steps. A prepared Streptavidin‐HRP solution was subsequently added, and substrate incubation was conducted using TMB. Following the termination of the reaction with the Stop solution, absorbance was measured at 450 nm, with a secondary measurement at 550 nm for correction purposes. IL‐6 concentrations were determined by comparing the absorbance values to a standard curve, demonstrating a sensitivity of less than 1 pg/mL and exhibiting high precision (CV < 10%).

### Assessment of oxidized low‐density lipoprotein (LDL_ox_)

The LDL_ox_ levels were measured utilizing the Human Oxidized LDL ELISA Kit (Abcam, ab285269) according to the manufacturer's instructions. Serum samples from controls, CAD patients, and candidates with ACS were initially diluted according to the protocol instructions. The diluted samples were subsequently loaded into the wells of a microtiter plate precoated with antibodies specific to LDL_ox_, along with the provided standards. Following an incubation period to facilitate binding, the wells were washed five times to eliminate unbound substances. A biotinylated detection antibody was then introduced, followed by the addition of streptavidin‐HRP, which facilitated the colorimetric reaction upon introducing TMB substrate. Following the subsequent incubation period, Stop solution was employed to terminate the reaction. Absorbance was then assessed at 450 nm using an ELISA plate reader. By comparing the absorbance values to the standard curve, the concentration of LDL_ox_ was calculated; these findings were then reported in pg/mL.

### Assessment of nitric oxide production

The NO Colorimetric Assay Kit (BioVision Inc., Milpitas, CA, USA, and MyBioSource) was used to quantify total nitrate and nitrite concentrations in biological samples through a two‐step enzymatic process. Initially, nitrate was enzymatically reduced to nitrite using nitrate reductase in conjunction with an appropriate enzyme cofactor. Subsequently, the resulting nitrite was reacted with Griess Reagents (Reagents 1 and 2), forming a deep purple azo compound, the absorbance of which was measured at a wavelength of 540 nm. A standard curve was constructed using known nitrate and nitrite standards, against which the absorbance of the samples was compared to ascertain their respective concentrations. Each well received up to 85 µL of the sample, with necessary adjustments made using Assay Buffer.

### Statistical analysis

The data distribution was assessed using the Shapiro–Wilk test. When the assumption of normality was not rejected (*p* value > 0.1), group comparisons were performed with a *t*‐test and one‐way ANOVA for three or more groups. If the assumption of normal distribution was rejected, the Mann–Whitney *U*‐test was used for two group comparisons, whereas the Kruskal–Wallis test was applied for more than two groups (Table 1). Values are presented as the median with interquartile range. Across independent groups, the chi‐square test was applied to compare the frequencies of the categorical variable. Correlation between two continuous variables was evaluated using Pearson correlation coefficient. Data are presented as the mean values ± SEM. *p* values were adjusted for multiple comparisons using the Tukey method. Fisher's exact test was used to analyze the proportions. *p* values are two‐sided and considered statistically significant if *p* < 0.05. All analyses were conducted using GraphPad Prism 8.

**Table 1:** Baseline characteristics of all participants

	No CAD	CAD	ACS	*p* value^a^	*p* value^b^	*p* value^c^	*p* Overall
*N*	90	90	90				
Age, years	63 (54–75)	70 (63–78)	68 (59–78)	**<0.001**	**0.02**	**0.36**	**0.003**
Male, no (%)	36 (40)	64 (71)	66 (73)	**<0.001**	**<0.001**	0.74	–
Hypertension, no (%)	65 (72)	87 (96)	76 (84)	**<0.001**	**0.047**	**0.005**	–
Diabetes, no (%)	10 (11)	36 (40)	35 (39)	**<0.001**	**<0.001**	0.88	–
Current smoking, no (%)	8 (9)	24 (27)	29 (32)	**0.002**	**<0.001**	0.41	–
Previous PCI/ACVB, no (%)	NA	36 (40)	17 (19)	–	–	**<0.001**	–
BMI, kg/m^2^	27 (24–32)	29 (26–32)	27 (25–31)	0.17	0.86	0.19	**0.024**
nHDL_ox_, no unit	0.58 (0.43–0.65)	0.72 (0.63–0.97)	1.4 (1.0–1.80)	**<0.001**	**<0.001**	**<0.001**	**<0.001**
HDL‐C, mg/dL	59 (52–72)	45 (40–55)	42 (35–52)	**<0.001**	**<0.001**	**0.01**	**<0.001**
LDL‐C, mg/dL	127 (97–152)	95 (74–123)	109 (80–150)	**<0.001**	**0.024**	0.035	**<0.001**
Cholesterol, mg/dL	195 (172–225)	171 (144–206)	178 (147–217)	**<0.001**	**<0.001**	0.16	**<0.001**
Triglyceride, mg/dL	115 (86–165)	128 (95–183)	134 (100–185)	**0.006**	**0.14**	0.34	**0.029**
Lipoprotein a, mmol/L	12 (6–34)	16 (7–84)	14 (5–44)	0.053	–	–	**0.05**
HbA1c, mmol/mol	5.6 (5.3–5.9)	5.9 (5.6–6.7)	5.8 (5.5–6.4)	**<0.001**	**<0.001**	**0.5**	**<0.001**

*Note*: Data are presented as median with interquartile range or number (%). Chi‐square test was used for comparison among the categorical variables and Fisher exact test for comparisons between groups. The Kruskal–Wallis test was used for continuous variables. If significant, the Mann–Whitney *U*‐test was used for pairwise comparisons. Statistically significant values (*p *< 0.05) are shown in bold numbers.

Abbreviations: ACVB, aorto‐coronary venous bypass; BMI, body mass index; CAD, coronary artery disease; HbA1c, glycated hemoglobin A1c; HDL‐C, high‐density lipoprotein cholesterol; LDL‐C, low‐density lipoprotein cholesterol; nHDL_ox_, lipid hydroperoxide content and redox activity of HDL normalized by HDL‐C levels; no, number; PCI, percutaneous coronary intervention.

^a^Comparison of no CAD versus CAD.

^b^Comparison of no CAD versus ACS.

^c^Comparison of CAD versus ACS.

## Results

### Demographic characteristics of study candidates

This study included 270 candidates. They were classified according to their coronary status (*n* = 90 in the CAD group, *n* = 90 in the no CAD group, and *n* = 90 with ACS). The characteristics of all study candidates are presented in Table 1. The incidence of CAD was significantly higher among candidates who were male, as well as those with hypertension, diabetes, or hyperlipidemia, compared to their female counterparts or those without the aforementioned risk factors (*p* < 0.001 for all comparisons). Furthermore, patients with CAD exhibited a higher prevalence of smoking behavior (*p* = 0.002). Additionally, patients with ACS were found to be slightly younger than those with stable CAD (*p* = 0.02), exhibited a lower frequency of hypertension (*p* = 0.047), and had higher LDL levels (*p* = 0.024) in comparison to the CAD group. Other risk factors, including diabetes mellitus and smoking, showed no significant differences among the groups.

### Antioxidant HDL function is reduced in CAD and ACS

The cohort of 180 patients diagnosed with CAD or ACS showed significantly elevated levels of oxidized high‐density lipoprotein (HDL_ox_) compared to the 90 patients without CAD (*p* < 0.001 for all comparisons, Table 1). Notably, participants who experienced an acute coronary event (*n* = 90) exhibited a median increase of 140% in oxidized HDL (nHDL_ox_) compared to the 90 patients without CAD (*p* < 0.001, Table 1). Among patients with CAD (*n* = 90), nHDL_ox_ levels were also significantly higher compared to those without CAD, with a median increase of 24%. Consequently, patients with ACS and CAD demonstrated reduced antioxidant HDL function when contrasted with participants without CAD. Furthermore, patients with ACS recorded the highest nHDL_ox_ values in all examined groups (*p* < 0.001, Table 1).

### Associations of HDL_ox_ and LDL_ox_ levels with markers of endothelial dysfunction and impaired HDL function in CAD and ACS

**Fig. 1:**
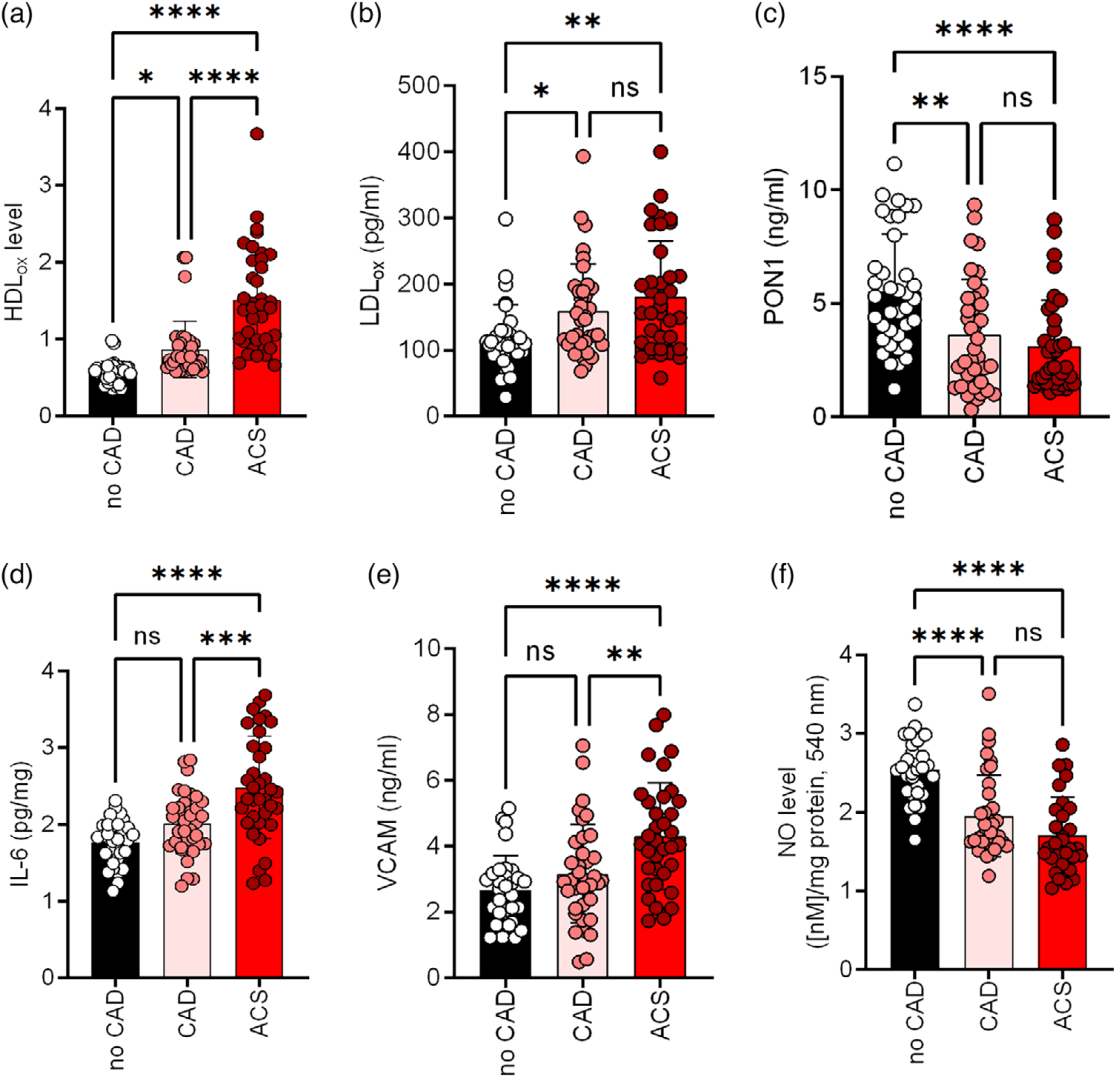
Levels of oxidative stress, inflammation, and endothelial dysfunction markers in patients with coronary artery disease and acute coronary syndrome compared to controls. This figure illustrates the levels of HDL_ox_: HDL‐lipid peroxide content (a), oxidized low‐density lipoprotein (LDL_ox_) (b), paraoxonase‐1 (PON‐1) (c), interleukin‐6 (IL‐6) (d), vascular cell adhesion molecule‐1 (VCAM‐1) (e), and nitric oxide (NO) bioavailability (f) in patients diagnosed with coronary artery disease (CAD) and acute coronary syndrome (ACS) in comparison to a control group. The data are presented as mean ± standard error of the mean (SEM) and analyzed using one‐way variance analysis (ANOVA). N = 30–37 samples per group; *p < 0.05, **p < 0.01, ***p < 0.001, ****p < 0.0001.

Fig. 1 illustrates the comparative levels of key biomarkers in patients diagnosed with CAD and ACS to the control group. The findings indicate that levels of HDL_ox_ were significantly elevated in both the CAD and ACS cohorts compared to the control group, with a clear difference between the CAD and ACS patients (Fig. 1a). LDL_ox_ was significantly elevated in both the CAD (159.3 ± 70.4 pg/mL) and ACS (180.7 ± 84.9 pg/mL) cohorts compared to the control group (117.5 ± 54.1 pg/mL, *p* = 0.001), with no notable difference observed between the CAD and ACS patients (Fig. 1b). Furthermore, PON‐1 activity was significantly diminished in both CAD (3.6 ± 2.4 ng/mL) and ACS (3.1 ± 2.0 ng/mL) groups compared to controls (5.5 ± 2.5 ng/mL, *p* < 0.0001), with no significant difference between the two patient groups (*p* = 0.38, Fig. 1c). IL‐6 levels were also significantly elevated in both CAD (2.0 ± 0.4 pg/mL) and ACS (2.5 ± 0.7 pg/mL) compared to controls (1.8 ± 0.3 pg/mL, *p* < 0.0001), with IL‐6 levels being significantly higher in patients with ACS than those with CAD (Fig. 1d). Additionally, VCAM‐1 levels were significantly increased in both CAD (3.2 ± 1.5 ng/mL) and ACS (4.3 ± 1.6 ng/mL) groups relative to controls (2.7 ± 1.0 ng/mL, *p *< 0.0001), with ACS patients exhibiting significantly higher VCAM‐1 levels than their CAD counterparts (Fig. 1e). Lastly, NO bioavailability was reduced in both CAD (1.95 ± 0.5 µM) and ACS (1.7 ± 0.5 µM) patients compared to controls (2.5 ± 0.4 µM, *p* < 0.0001); however, no significant difference was noted between the CAD and ACS groups (*p* = 0.24, Fig. 1f). These findings underscore substantial alterations in biomarkers associated with inflammation, endothelial dysfunction, and oxidative stress in patients with CAD and ACS.

### Correlations of HDL_ox_ levels with markers of endothelial dysfunction and impaired HDL function in control, CAD, and ACS groups

**Fig. 2:**
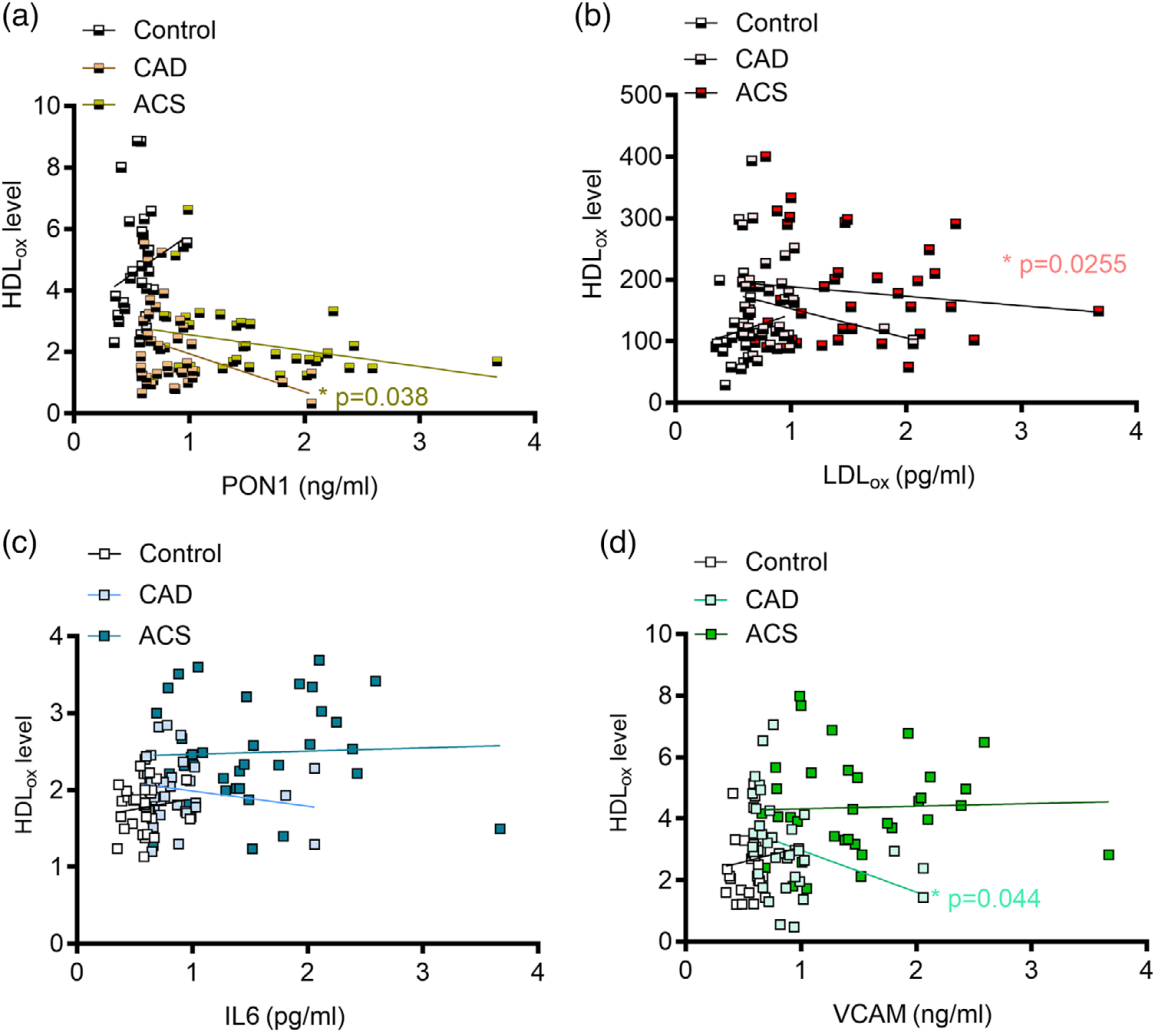
Correlation analysis of biomarkers associated with oxidative stress, inflammation, and endothelial dysfunction in control, CAD, and ACS cohorts. Scatter plots illustrate the correlation between lipid, inflammatory, and endothelial dysfunction biomarkers across control, coronary artery disease (CAD), and acute coronary syndrome (ACS) cohorts. Pearson correlation coefficients were computed to evaluate the relationships among (a) HDL_ox_ and paraoxonase‐1 (PON‐1) activity, (b) HDL‐lipid peroxide content and oxidized low‐density lipoprotein (LDL_ox_), (c) HDL_ox_ and interleukin‐6 (IL‐6), (d) HDL_ox_ and vascular cell adhesion molecule‐1 (VCAM‐1). The data are presented as mean ± standard error of the mean (SEM) and analyzed using one‐way variance analysis (ANOVA); n = 30–37 samples per group; *p < 0.05.

Fig. 2a reveals a significant negative correlation between oxidized HDL (HDL_ox_) and PON‐1, particularly within the CAD group (*ß* 0.57, 95% CI −2.4 to −0.07, *p* = 0.03), indicating that higher HDL_ox_ levels are associated with lower PON‐1 activity. Fig. 2b shows no significant correlation between HDL_ox_ and LDL_ox_ across all groups (*p* = ns). Fig. 2c illustrates the absence of a significant correlation between HDL_ox_ and IL‐6 in all groups (*p* = ns). Fig. 2d indicates a significant positive correlation between HDL_ox_ and VCAM exclusively in the CAD group (*ß* 0.65, 95% CI −2.6 to −0.03, *p* = 0.04), where increased HDL_ox_ levels correlate with elevated VCAM levels. These findings highlight specific relationships between lipid oxidation, endothelial dysfunction, and inflammation, especially in the context of CAD.

### Correlation analysis of lipid, inflammatory, and endothelial dysfunction biomarkers in CAD and ACS

**Fig. 3:**
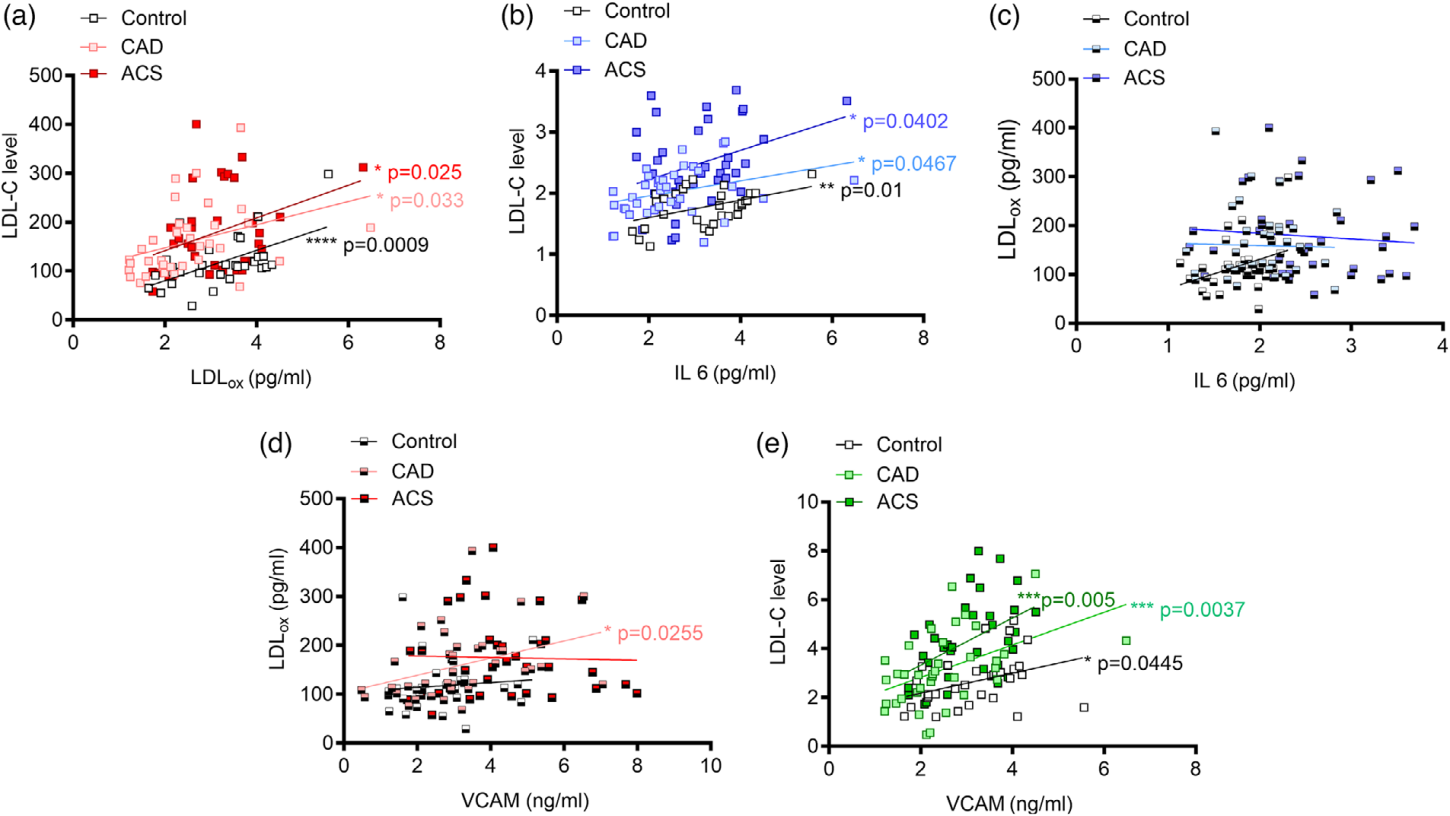
Correlation analysis of lipid, inflammatory, and endothelial dysfunction biomarkers in control, CAD, and ACS cohorts. Scatter plots illustrate the correlation between lipid, inflammatory, and endothelial dysfunction biomarkers across control, coronary artery disease (CAD), and acute coronary syndrome (ACS) cohorts. Pearson correlation coefficients were computed to evaluate the relationships among (a) low‐density lipoprotein (LDL) and oxidized low‐density lipoprotein (LDL_ox_), (b) LDL level and interleukin‐6 (IL‐6), (c) LDL_ox_ and vascular cell adhesion molecule‐1 (VCAM‐1), (d) LDL_ox_ and VCAM‐1, (e) LDL level and VCAM‐1. The data are presented as mean ± standard error of the mean (SEM) and analyzed using one‐way variance analysis (ANOVA); *n = 30–37 samples per group; p < 0.05, **p < 0.01, ***p < 0.001, ****p < 0.0001.

Fig. 3 presents a correlation analysis among various biomarkers in the control, CAD, and ACS groups. Fig. 3a illustrates a significant positive correlation between LDL and oxidized LDL across all groups, demonstrating that increased LDL levels correspond to elevated LDL_ox_ levels in control (*ß* 8.4, 95% CI 14.0–48.2, *p* < 0.001), CAD (*ß* 10.6, 95% CI 1.9–45.1, *p* = 0.05), and ACS (*ß* 14.3, 95% CI 4.3–62.5, *p* = 0.02) patients. Fig. 3b depicts a significant positive correlation between LDL and IL‐6 across all groups, indicating that higher LDL levels are associated with increased IL‐6 levels in control (*ß* 0.05, 95% CI 0.03–0.24, *p* = 0.01), CAD (*ß* 0.06, 95% CI 0.02–0.24, *p* = 0.04), and ACS (*ß* 0.01, 95% CI 0.01–0.47, *p* = 0.04) patients. Fig. 3c shows no significant correlation between IL‐6 and LDL_ox_ across all groups (*p* = ns). Fig. 3d indicates a significant positive correlation between LDL_ox_ and VCAM solely in the CAD group (*ß* 7.5, 95% CI 50.9–157.1, *p* = 0.02), where increased LDL_ox_ levels correlate with elevated VCAM levels, and higher IL‐6 levels are associated with increased LDL_ox_ levels within this cohort. Fig. 3e reveals a similar significant correlation between LDL and VCAM across all groups, wherein elevated LDL levels relate to higher VCAM levels in the control (*ß* 0.2, 95% CI 0.01–0.8, *p* = 0.04), CAD (*ß* 0.21, 95% CI 0.23–1.0, *p* = 0.003), and ACS groups (*ß* 0.32, 95% CI 0.31–1.7, *p* = 0.005). These findings underscore specific relationships between lipid and inflammatory markers, particularly in the context of CAD.

## Discussion

In the present study, we investigated the association of oxidized HDL as an instigator of impaired HDL function with key markers of vascular health. We found that patients with ACS and CAD had elevated HDL lipid peroxide (nHDL_ox_) levels as a measure of decreased HDL antioxidant function compared to participants with no CAD. High levels of HDL_ox_ were further associated with decreased PON‐1 activity (a key enzyme in HDL‐mediated antioxidant function), higher levels of oxidized LDL (LDL_ox_), and increased VCAM‐1, all of which contribute to endothelial dysfunction and atherogenesis. However, our results did not show any significant correlations between HDL_ox_ levels and key markers of vascular health (PON‐1 activity, VCAM‐1, LDL_ox_) in participants with ACS. One possible explanation for this finding might be a delayed effect of increased HDL_ox_ levels on vascular markers, which may not yet be measurable in the very acute onset of an acute cardiac event. This hypothesis is supported by our further findings that increased HDL_ox_ levels are present in CAD patients, whereas at the same time, significant correlations between HDL_ox_ levels and VCAM‐1 levels and PON‐1 activity can be found in the CAD group. It is conceivable that the biological consequences of HDL_ox_ and the effects arise with different dynamics and are measurable at different time points. As shown in our previous study, increased levels of oxidized HDL were identified in CAD and ACS patients; however, the absence of key vascular markers limited the insight into potential causal pathways [18]. The present findings advance this work by establishing a mechanistic link between oxidized HDL and acute coronary events. However, further studies are necessary to demonstrate the temporal correlation between oxidized HDL and vascular markers investigated in the present study.

Beyond our findings regarding HDL_ox_, our study revealed that a decreased PON‐1 activity is a significant characteristic in patients with CAD and ACS, notably associated with elevated levels of LDL_ox_ and VCAM‐1. PON‐1 plays a pivotal role in modulating HDL function, particularly its antioxidative capacity, and its reduced activity underscores the impairment of HDL's protective functions [31]. The decline in PON‐1 activity, coupled with the increased levels of oxidized LDL and oxidized HDL, suggests a disruption in the balance between pro‐oxidative and antioxidative forces, which may exacerbate the pathogenesis of atherosclerosis [32]. Furthermore, the upregulation of VCAM‐1, observed in both the CAD and ACS cohorts, further implicates inflammation as a driving factor in atherosclerotic plaque formation and progression [33, 34]. These findings highlight the intricate interplay between oxidative stress, inflammation, and lipid metabolism in the development of CVDs, suggesting that strategies aimed at restoring PON‐1 activity and reducing LDL_ox_ may hold therapeutic potential in mitigating atherosclerosis‐related complications [35].

Furthermore, our results suggest that HDL_ox_ is not only linked to the mere presence of atherosclerosis but also with the severity and activity of the disease, as varying levels of HDL_ox_ are observed at different stages of ischemic heart disease. In particular, the highest levels of HDL_ox_ were identified in patients with ACS, underscoring its role in acute disease activity. This finding contrasts with patients exhibiting stable CAD who also demonstrate elevated HDL_ox_ levels when compared to healthy controls. These observations imply that the oxidative modification of HDL occurs early in the disease process, potentially serving as a biomarker for disease progression. However, as mentioned above, the effects accompanying elevated HDL_ox_ levels on vascular health markers may be measurable at different time points, particularly in the case of acute cardiovascular events.

Additionally, our study revealed a significant association between PON‐1 activity and VCAM‐1 expression, particularly in CAD patients. The observed reduction in PON‐1 activity in both CAD and ACS patients correlated with increased VCAM‐1 levels, suggesting a potential link between impaired HDL antioxidant function and endothelial dysfunction. VCAM‐1, a key adhesion molecule involved in leukocyte recruitment, is upregulated in response to the pro‐inflammatory environment created by dysfunctional HDL [36, 37]. Oxidized HDL disrupts its interaction with the SR‐B1 receptor, leading to the loss of its anti‐inflammatory properties and the acquisition of pro‐inflammatory functions [26, 27]. This dysregulation plays a central role in atherogenesis, as VCAM‐1 facilitates leukocyte adhesion, migration, and extravasation across the vascular endothelium [38]. Although functional HDL suppresses VCAM‐1 expression in endothelial cells, oxidized HDL paradoxically enhances its expression, particularly in the presence of TNF‐α, resulting in a nearly five‐fold increase in surface VCAM‐1 levels [16]. This upregulation, in combination with NF‐κB activation, exacerbates endothelial dysfunction and promotes leukocyte recruitment, further accelerating atherogenesis.

Although significant associations were observed between HDL_ox_ and markers such as PON‐1 and VCAM‐1 in the CAD group, it is important to note that several relationships were not statistically significant in multivariate analyses, particularly in the ACS group. For example, HDL_ox_ did not correlate significantly with IL‐6 or LDL_ox_, and associations with VCAM‐1 and PON‐1 were absent in ACS patients. These non‐significant findings may reflect the temporal dynamics of acute coronary events, where oxidative and inflammatory markers fluctuate rapidly and may not yet reflect stable mechanistic relationships. Multiple studies have shown that levels of hs‐CRP, IL‐6, and other endothelial activation markers show dynamic changes following an acute cardiovascular event, with peaks and declines influenced by timing of sample collection, clinical course, and therapeutic interventions [39, 40, 41, 42]. Therefore, inflammatory and oxidative biomarkers are known to exhibit significant temporal variability, especially in the context of ACSs. The lack of significant associations in our ACS subgroup may reflect a temporal mismatch between the inflammatory state and the oxidative characteristics of HDL, rather than a true absence of pathophysiological relevance. Alternatively, it could point to differential pathophysiologic pathways active in chronic versus acute disease states that require further investigation.

Oxidized HDL disrupts oxidative stress homeostasis, a key driver of atherosclerosis. In its functional state, HDL counteracts oxidative stress by scavenging ROS and preventing the oxidation of LDL, thereby supporting vascular health and reducing the risk of CVD [43]. This protective function is primarily attributed to antioxidant enzymes, particularly PON‐1. However, when HDL undergoes oxidative modification, its capacity for cholesterol efflux is reduced, and its effectiveness at inhibiting LDL oxidation diminishes, regardless of its concentration [15]. Patients with ACS [44] often exhibit oxidized HDL with diminished PON1 activity. However, we found that the reduction in PON1 activity was significantly associated with higher HDL_ox_ levels in both CAD and ACS groups, reinforcing the detrimental effects of oxidative modification of HDL. The findings of our study suggest that diminished PON‐1 activity, in conjunction with increased LDL_ox_ levels and VCAM‐1, may promote endothelial activation, thereby accelerating atherosclerotic plaque formation and contributing to the progression of CVD.

Our study highlights the dual role of oxidized HDL in CAD pathophysiology—both as a marker of oxidative stress and as a mediator of inflammation through VCAM‐1 upregulation. This underscores its potential as a therapeutic target for mitigating chronic inflammation and slowing disease progression. Notably, previous studies have demonstrated that oxidized HDL contributes directly to atherogenesis in an ex vivo mechanistic assay of atherogenesis [29]. HDL_ox_ has also been validated against various methods for measuring HDL function, including the HDL inflammatory index, cholesterol efflux capacity, and antioxidative capacity [29]. We now have additionally performed measurements of several markers of vascular health, including PON‐1 activity, VCAM‐1 levels, NO production, and LDL_ox_. These measurements reflect different components of HDL function. We aimed to gain insights into the mechanisms underlying atherosclerosis and CVD by assessing these markers. Given the controversial evidence regarding the clinical relevance of HDL function, our findings align with the hypothesis that oxidation of HDL is a strong predictor of atherosclerosis and, importantly, of acute coronary events. The cell‐free fluorometric method utilized in our study to measure HDL_ox_ has been previously applied in several studies of atherosclerotic CVD [18, 23, 29, 45, 46, 47, 48, 49], and it was recently proposed as a diagnostic biomarker for CAD [50]. As lipid peroxidation is recognized as a significant contributor to the inflammation of arterial walls [1, 16, 51], the redox properties of HDL may serve as a potential marker for CVD and related biological processes in humans [23, 29]. We observed that HDL_ox_ levels are highest in patients with ACS, whereas patients with stable CAD still have higher levels than healthy controls. Correspondingly, the parameters of HDL function measured in this study, which all indicate a loss of HDL's atheroprotective function, correlate with these findings. Thus, our study is the first published investigation to establish a mechanistic pathway linking HDL_ox_ to the presence of an ACS.

Our study has several limitations. The cross‐sectional design prevents us from establishing causal relationships between oxidized HDL (HDL_ox_) levels and the progression of CAD and ACS. Longitudinal studies are needed to determine whether increased HDL_ox_ levels precede or result from disease progression. This is particularly important, as the temporal correlation between oxidized HDL, the investigated vascular markers, and functional outcomes is unknown, especially in the case of acute cardiovascular events. In this context, selection bias is another potential limitation, particularly due to patient recruitment during elective or urgent coronary angiography, which may favor patients with more advanced or severe disease. Furthermore, our study design may limit generalizability, as participants were recruited from two university hospitals in Germany and may not reflect broader populations with varying ethnicities, healthcare access, or environmental exposures. Although we adjusted for key clinical covariates, residual confounding cannot be excluded. In particular, medication use—including statin therapy—may have influenced the inflammatory and oxidative biomarkers analyzed. Although our previous findings [18] demonstrated that statins do not significantly affect HDL antioxidant function in CAD patients, the potential influence of other medications as well as unmeasured factors such as diet, physical activity, or specific HDL subfractions cannot be excluded. Finally, the possibility of reverse causality in the relationship between HDL_ox_ and acute coronary events represents another limitation for our investigation. Although our findings indicate a significant association between elevated levels of HDL_ox_ and an impaired cardiometabolic function, it is crucial to acknowledge that this relationship may not be entirely causal. Future studies incorporating mechanistic experiments or interventional studies targeting HDL functionality could further clarify these relationships.

In conclusion, this cross‐sectional study provides further evidence regarding the pivotal role of dysfunctional HDL in atherogenesis. Oxidized HDL is associated with an impaired cardiometabolic function as expressed by increased VCAM, LDL_ox_, and decreased PON‐1 activity. Patients with ACS had the highest serum concentrations of HDL_ox_, surpassing those with stable CAD or healthy participants, independent of classical cardiovascular risk factors. Similarly, patients with ACS showed significantly higher VCAM and LDL_ox_ levels than patients with CAD or controls. These findings support the hypothesis that reduced antioxidant HDL function is critical in the progression from subclinical atherosclerosis to manifested CAD. Furthermore, they highlight new potential therapeutic targets aimed at restoring HDL functionality to mitigate disease progression.

## Conflict of interest statement

The HDL_ox_ assay is associated with the patent PCT/US2015/018147 (to TK). The co‐authors declare no conflict of interest.

## Funding information

This research was supported by grants from Brandenburg Medical School Theodor Fontane and from the Stiftungsfond BIOX (to N.P.). This research received partial support from NIH grants R01AG059501 (to T.K.). This research was also supported by the European Union's Horizon 2020 research and innovation program under grant agreement number 739593 (to N.H.), Deutsche Forschungsgemeinschaft HA 7512/2‐4 and HA 7512/2‐1 (to N.H.) and Innovation Forum program of the Medical Faculty number IF‐023‐22 and IF‐034‐22 (to N.H. an I.E.B.). I.E.B. obtained support from the German Heart Foundation, Else‐Kröner‐Fresenius foundation, and Hector Foundation.

## Ethics statement

All participants provided written informed consent. The study received approval from the local ethics committees of the Medical Association of Brandenburg (Nr. AS69bB/2016) and of the Ruhr University of Bochum (Nr. 15–5279) in compliance with the Declaration of Helsinki.

## Data Availability

The data supporting the findings of this study can be obtained from the corresponding author upon reasonable request.
